# The Effect of Overexpressed DdRabS on Development, Cell Death, Vesicular Trafficking, and the Secretion of Lysosomal Glycosidase Enzymes

**DOI:** 10.3390/biology7020033

**Published:** 2018-05-28

**Authors:** Azure Yarbrough, Katherine Maringer, Entsar J. Saheb, Sanaa Jawed, John Bush

**Affiliations:** 1 Biology Department, University of Arkansas at Little Rock, 2801 South University Ave, Little Rock, AR 72204-1099, USA; kvmaringer@hotmail.com (K.M.); ejsaheb@ualr.edu (E.J.S.); stjawed@ualr.edu (S.J.); jmbush@ualr.edu (J.B.); 2 Otsuka Pharmaceuticals, Greater Pittsburg Area, Pittsburgh, PA 15201, USA; 3 Faculty Member of Biology Department, Baghdad University, Baghdad 10071, Iraq

**Keywords:** *Dictyostelium*, RabS, vesicular trafficking, lysosomal enzymes, β-glucosidase, GTPase, development, cell death

## Abstract

Rab GTPases are essential regulators of many cellular processes and play an important role in downstream signaling vital to proper cell function. We sought to elucidate the role of novel *D. discoideum* GTPase RabS. Cell lines over-expressing DdRabS and expressing DdRabS N137I (dominant negative (DN)) proteins were generated, and it was determined that DdRabS localized to endosomes, ER-Golgi membranes, and the contractile vacuole system. It appeared to function in vesicular trafficking, and the secretion of lysosomal enzymes. Interestingly, microscopic analysis of GFP-tagged DdRabS (DN) cells showed differential localization to lysosomes and endosomes compared to GFP-tagged DdRabS overexpressing cells. Both cell lines over-secreted lysosomal glycosidase enzymes, especially β-glucosidase. Furthermore, DdRabS overexpressing cells were defective in aggregation due to decreased cell–cell cohesion and sensitivity to cAMP, leading to abnormal chemotactic migration, the inability to complete development, and increased induced cell death. These data support a role for DdRabS in trafficking along the vesicular and biosynthetic pathways. We hypothesize that overexpression of DdRabS may interfere with GTP activation of related proteins essential for normal development resulting in a cascade of defects throughout these processes.

## 1. Introduction

*Dictyostelium discoideum* is a haploid social amoeba that has proven to be an excellent model system for studying a number of processes including adhesion, cell–cell signaling, development, host pathogen interactions, and gene regulation among others. It has three life cycles consisting of a vegetative phase, a developmental phase, and a sexually reproductive phase [1]. Under normal conditions, *D. discoideum* are unicellular amoeba, but upon starvation, the cells begin to secrete cAMP in oscillating waves causing them to form multicellular aggregates that move toward the increasing concentration of cAMP. Within 24 h of the onset of development, the aggregates form mounds, slugs, and finally, a stalk of vacuolated cells and a fruiting body that contains thousands of spore cells; the stalk cells are a result of a type of programmed cell death, and the spores will germinate into amoeba [2]. A variety of proteins such as adhesion proteins, G proteins, second messengers, surface receptors, prespore markers, and prestalk markers are involved in this process, and while much is known about the types of proteins that are secreted or are involved in adhesions during development, the mechanism of their secretion and delivery to the cell surface is unknown [3]. The movement of these proteins appears to rely on vesicular trafficking suggesting the involvement of Rab GTPases [3,4,5].

Rab proteins belong to the family of small molecular weight GTPases; they are evolutionarily conserved essential regulators of membrane trafficking [6]. They act as molecular switches that cycle between an inactive GDP- and an active GTP-bound state [7]. Rabs are essential for signaling and the control of cell proliferation and differentiation. Since they are found downstream in signaling cascades, they can impact gene expression and influence growth [6] They serve as scaffolds to temporally and spatially integrate membrane trafficking and intracellular signaling [8,9]. Rabs are best known for their roles in exocytic and endocytic membrane trafficking, which encompasses the constitutive and regulated secretory routes, endocytosis via caveolae or clathrin-coated vesicles (CCVs), micropinocytosis, and phagocytosis. They can control anterograde and retrograde trafficking between cellular compartments to coordinate cargo delivery and membrane recycling [6], and they have been implemented in the control of cell-type-specific functions, such as regulated secretion [6].

*Dictyostelium* has proven to be a useful system in which to investigate endosomal and lysosomal membrane trafficking [10,11]. It is a professional phagocyte, and pinocytosis occurs at a very high rate in *Dictyostelium;* fluid-phase nutrients are internalized by macropinocytosis, concentrated in endosomes, and degraded in lysosomes [10,11,12]. This can easily be manipulated through the size of the particles used; fluid-phase endocytosis is involved with the up-take of non-particulate materials like RITC-dextran, while phagocytosis can be triggered by using larger particles, such as latex beads. Lysosomes connect at least three membrane trafficking pathways including the endocytic, biosynthetic, and phagocytic pathways [10,11,13] all of which have been extensively studied. The biosynthetic pathway includes the biosynthesis of lysosomal enzymes; these are synthesized in the ER as membrane-bound, N-glycosylated precursor proteins, and then they are transported to the Golgi. Lysosomal enzymes are targeted to lysosomes. However, the recognition and sorting machinery involved in this part of the process is poorly characterized in *Dictyostelium*. Unlike mammalian cells, *D. discoideum* appear to lack the mannose 6-phosphate receptors (MPRs) used in the targeting of lysosomal enzymes suggesting the existence of another lysosomal enzyme receptor [10,11,14].

It was previously observed that DdRabS localizes to ER-Golgi membranes and the contractile vacuole system [15]. This protein is a Rab GTPase that is somewhat homologous to Rab1 in other organisms; as is commonly done in this organism, proteins with a lower degree of homology are typically given a letter as a name while those with higher homology use a number and additional letters for any existing isoforms. In this study we report an investigation into the role of RabS in *Dictyostelium* development, endocytosis, endocytic recycling, and the secretion of lysosomal enzymes. We have found that these Rab proteins can play multiple roles in the cell, so numerous studies were undertaken. Overexpressing DdRabS cells showed an increase in the rate of phagocytosis and pinocytosis with a corresponding increase in growth rate. It was observed that DdRabS were unable to complete the developmental process as they could not develop fruiting bodies. Aggregation was severely defective as cells had decreased cell-cell cohesion and chemotactic response to cAMP. Furthermore, when incubated with DIF-1 to induce cell death, DdRabS over-expressing cells showed a significant increase in cell mortality. Interestingly, cells expressing a dominant negative form of the DdRabS protein behaved similarly to the wild type (WT) AX4 cells. This data suggests that overexpression of RabS causes a cascade of signaling defects crucial to normal development in *Dictyostelium*.

## 2. Materials and Methods

### 2.1. Cells and Culture Conditions

All *D. discoideum* cell lines, WT strain AX4, pDneo2a-GFP, DdRabS-GFP, and DdRabS(DN)-GFP were grown axenically at 21 °C in shaking culture at 150 r.p.m. in HL5 medium:1% oxoid proteose peptone, 1% glucose, 0.5% yeast extract (Fisher Biotech, Fair lawn, NJ, USA), 2.4 mM Na_2_HPO_4_, and 8.8 mM KH_2_PO_4_, pH 6.5, and the media was supplemented with 300 mg/mL of streptomycin sulfate and 100 mg/mL of ampicillin (Sigma, St. Louis, MO, USA). Transfected cells also received 10 mg/mL of G418 (Invitrogen, Grand Island, NY, USA). To minimize autofluorescence from the media, cells were incubated in LoFlo media : 0.1% FM salts 1 (500 mM NH_4_Cl 200 mM MgCl_2_ 10 mM CaCl_2_), 0.1% FM salts 2 (FeCl_3_ 50 mM), 0.01% FM trace elements (8.56 mM Na_2_-EDTA·2H_2_O, 13 mM ZnSO_4_·H_2_O, 14.5 mM H_3_BO_4_, 2.6 mM MnCl_2_·4H_2_O, 0.7 mM CoCl_2_·6H_2_O, 0.6 mM CuSO_4_·5H_2_O, 0.1 mM (NH_4_)6Mo_7_O_24_·4H_2_O pH 6.5), 63 mM glucose, 5 mM K_2_HPO_4_, 1% casein peptone, pH 6.5 for 24 h before fluorescent experiments (www.dictybase.org).

### 2.2. Creation of GFP Tagged DdRabS Cell Lines

The DdRabS overexpressing cell line was created by amplifying the gene from genomic DNA using polymerase chain reaction. The resulting amplificon was ligated into the pDneo2A-GFP vector and transformed into the AX4 strain of *Dictyostelium discoideum* using electroporation; transformants were selected for using the antibiotic G418 (Invitrogen, Grand Island, NY, USA. The DdRabS(DN) cell line was created using the Stratagene QuickChange^®^ II Site-Directed Mutagenesis Kit (Stratagene, Santa Clara, CA, USA). The resulting plasmid was also transformed into AX4 and selected for with G418 [15].

### 2.3. Endocytosis Assays

Phagocytosis was measured by incubating 10 mL of 2 × 10^6^ cells/mL with 100 µL of fluorescent 2 µm rhodamine isothiocynate labeled latex beads (200:1 beads per cell) (RITC- latex beads, Sigma Aldrich, St. Louis, MO, USA) on a rotary shaker, and 1 mL samples were taken at the indicated time points (0, 15, 30, 45, 60 min); 100 µL were taken at the 0 time point and tested for protein content by Bradford assay. Samples were placed into 2 mL ice-cold Soerensen buffer (SB): 14.5 mM KH_2_PO_4_, 2.5 mM Na_2_HPO_4_, pH 6, and layered on top of 10 mL 20% *w/v* PEG 8000. Then they were centrifuged at 800 *g* and 4 °C for 10 min; the cell pellet was washed twice with cold SB, resuspended in 1 mL lysis buffer (50 mM Na_2_HPO_4_, pH 9.3, 0.2% Triton X-100), and fluorescence was measured with a Horiba Jobin Yvon analytical grade FluorMax3 Spectrofluorimeter, excitation 544 nm, emission 574 nm. Relative fluorescence was plotted in comparison to the WT as a function of time after correcting for differences in protein content. Experiments were performed in triplicate.

Fluid phase pinocytosis was measured by incubating 5 mL cells at 5 × 10^6^ cells/mL with 100 µL of 100 mg/mL fluorescent rhodamine isothiocynate dextran, molecular weight 10,000, (RITC- dextran, Sigma Aldrich, St. Louis, MO, USA) on a rotary shaker, and 500 µL samples were taken at the indicated time points (0, 15, 30, 45, 60, 90, 120, 180 min); 100 µL of the cell suspension was tested for protein content by Bradford assay. Samples were placed into 50 µL of Trypan blue, inverted once, and centrifuged at 800 *g* 4 °C for 2 min. The supernatant was discarded, the pellet was washed with 1 mL SB, and then resuspended with 1 mL SB. Fluorescence was measured with a Horiba Jobin Yvon analytical grade FluorMax3 Spectrofluorimeter, excitation 544 nm, emission 574 nm. Fluorescence was plotted against time after subtracting the fluorescence at the 0 min time point and correcting for differences in protein concentration. Experiments were performed in triplicate.

Exocytosis was measured by preparing the cells as for the pinocytosis assay; after 3 h, the cells were centrifuged at 500 *g* for 3 min, washed twice with nutrient media, and resuspended in 5 mL fresh media (RITC-dextran, Sigma Aldrich, St. Louis, MO, USA). Then 500 µL samples were taken at the indicated time points and treated as in the pinocytosis assay (0, 15, 30, 45, 60, 90, 120, 180 min). Fluorescence was measured with a Horiba Jobin Yvon analytical grade FluorMax3 Spectrofluorimeter, excitation 544 nm, emission 574 nm. Fluorescence was plotted against time; the 0 min time point for each cell line was assigned 100% relative fluorescence, and the rest of the time points were calculated relative to them. Experiments were performed in triplicate.

Recycling rates were measured by incubating 10^8^ cells in 4 mg Lucifer yellow (Lucifer yellow, Sigma Aldrich, St. Louis, MO, USA) dissolved in 10 mL of medium; then, the cells were centrifuged at 500 *g* for 3 min and resuspended in LoFlo medium. Then, 1 mL samples were taken at the indicated time points and added to 100 µL of Trypan blue, inverted, and centrifuged for 2 min at 800 *g*. The pellets were resuspended in 1 m SB, and the fluorescence was measured with a Horiba Jobin Yvon analytical grade FluorMax3 Spectrofluorimeter, excitation 470 nm, emission 515 nm. Again, the fluorescence at the 0 time point was subtracted from each data point and then plotted against time. The 0 min time point for each cell line was assigned 100% relative fluorescence, and the rest of the time points were calculated relative to them [16]. Experiments were performed in triplicate.

### 2.4. Endosome Visualization: RITC-Dextran Loading

Rhodamine B isothiocynate-dextran (RITC-dextran, Sigma Aldrich, St. Louis, MO, USA) is a fluid that is internalized but not degraded by endosomes [17]. Cells were harvested, placed in a 35 × 10 mm Petri dish containing 2 mL of HL5 medium, and supplemented with 40 µL of 100 mg/mL RITC-dextran; the cells were incubated for 60 min, washed with fresh media, and allowed to settle on a glass coverslip. All cell lines were photographed using the BrightLine^®^ TXRED filter set to visualize endosomes and the BrightLine^®^ GFP filter set was used to visualize GFP fluorescence on a Nikon 2000SE microscope with IPLab 3.7 software at 100× magnification. Quantification was performed using Fiji by ImageJ. The Pearson’s Correlation Coefficient (PCC) was reported.

### 2.5. Growth Rate

All cell lines were grown in HL5 medium until a density of 1–3 × 10^4^ cells/ mL was obtained. Cellular growth was determined by counting the cells every 24 h over five days using a hemacytometer (Hausser Scientific, Horshman, PA, USA). Experiments were performed in triplicate.

### 2.6. Cell Size and Diameter

Cell size was determined by taking cells in exponential growth phase, washed in SB, and then resuspended in equal volumes of SB. Then, they were placed on a microscope slide. 50 cells were photographed and measured using a Nikon 2000SE microscope with IPLab 3.7 software (Scanalytics, Inc., Fairfax, VA, USA) with 100× magnification. Experiments were performed in triplicate.

### 2.7. Lysosome Visualization: LysoTracker^®^ Blue Staining

LysoTracker^®^ Blue (Molecular Probes) was used to mark acidic organelles like lysosomes [18]. Cells were harvested and allowed to adhere to coverslips; the medium was aspirated, replaced with fresh medium containing 100 nM LysoTracker^®^ Blue, and incubated for 30 min at room temperature. The cells were washed twice with fresh media, and fluorescence was photographed with a Nikon 2000SE microscope with IPLab 3.7 software (Scanalytics, Inc., Fairfax, VA, USA) with 100× magnification using the BrightLine^®^ DAPI filter set, causing lysosomes to fluoresce blue, and the BrightLine^®^ GFP filter set was used for comparison with GFP localization. Quantification was performed as previously described.

### 2.8. Secretion of Lysosomal Enzymes under Starvation Conditions

The standard secretion assay was performed as described by [19]. Log phase cells were harvested by centrifugation, resuspended at a concentration of 5 × 10^7^ cells/mL in starvation buffer (10 mM phosphate buffer, pH 6.0), and incubated at 21 °C; samples were removed at the indicated time points (0, 15, 30, 60, 90, 120, 180, 280 min), and enzyme assays were performed on the supernatant (extracellular) and cell pellets (intracellular). The α-mannosidase, N-acetylglucosaminidase, β-galactosidase, acid phosphatase, and β-glucosidase activity was measured as previously described [19,20]. The 0 min time point for each cell line was assigned as the 100% relative amount, and the rest of the time points were calculated relative to them. Experiments were performed in triplicate.

### 2.9. Developmental Assay

Cells were grow axenically in HL5 medium on a rotary shaker at 160 r.p.m. and 1 × 10^9^ cells/ mL were harvested by centrifugation. After washing three times with developing buffer (5 mM Na_2_HPO_4,_ 5 mM Na_2_HPO_4,_ 5 mM KH_2_PO_4,_ 1 mM CaCl_2,_ 2 mM MgCl_2_), cells were resuspended at a density of 2 × 10^8^ cells/m; 200 µL of the cell suspension was spread evenly on a 100 mm KK_2_ plate using a sterile glass spreader. The plates were wrapped with a wet paper towel, sealed in plastic wrap, inverted, and incubated at 22 °C. After 6 h, pictures were taken every 2 h to monitor the different developmental stages; this method was adapted from www.dictybase.org. Photographs of multicellular development were taken using a model AM 4201, 25× microscope (Lutron Instruments, Tamil Nadu, India).

### 2.10. Cell Cohesion Assay

Cell cohesion assays were performed as described by [21,22,23]. To determine developmental cell cohesion, vegetative cells were centrifuged at 700 *g* for 4 min, washed in KK_2_, resuspended in KK_2_ at 2 × 10^7^ cells/mL, and starved in rotary-agitated suspension (175 r.p.m.) at 22 °C for 4 h. The cell suspension was diluted to a density of approximately 2.5 × 10^6^ cells/mL, and aggregates were dispersed by vigorously vortexing for 15 s. Aggregates were allowed to reform while rotating on a platform shaker at 180 rpm at room temperature. At the indicated times (the number of non-aggregated cells, including singlets and doublets, were scored using a hemocytometer; the number of aggregating cells was determined by subtracting this number from the total number of cells and was expressed as a percentage of the total.

To assess EDTA-resistant cell-cell adhesion, cells were harvested and resuspended in KK_2_, starved in a rotary shaker at 22 °C for 4 h, centrifuged, and resuspended in KK_2_ + 10 mM EDTA; this buffer inhibits EDTA-sensitive cohesion. Aggregates were dissociated by brief vortexing, and samples (0.2 mL) were placed in plastic tubes and rotated vertically at 180 rpm at room temperature for cell re-association. The percentage of cell aggregation was monitored at regular intervals for 60 min. Experiments were performed in triplicate.

### 2.11. Chemotaxis in Response to cAMP

Cell lines were grown axenically in HL5 medium and 1 × 10^7^ cells were harvested by centrifugation. Cells were washed twice with developing buffer and resuspended at a density of 5 × 10^6^ cells/ mL. After shaking for 8 h to allow allowing for the development of the cAMP receptor, 100 µL of the cell suspension was added to an outer 2 mm trough in a 60 mm agarose plate. At the same time, 100 µL of 5 µM cAMP was added to the center trough. Images were taken every h for 4 h using a Nikon 2000SE microscope with NIB Image software at 10× magnification. The edge of the trough was included in the image as a reference point.

### 2.12. Flow Cytometry Assay

To induce cell death, vegetative cells in late exponential growth phase were harvested and washed twice with SB. Approximately 5 × 10^5^ cell/ mL were suspended in 1 mL of SB containing 3 mM cAMP (Sigma) in a flask and incubated for 8 h at 22 °C. Cells were washed with 1 mL of SB which was replaced afterward with either 1 mL of SB or 1 mL of SB containing 0.1 mM differentiation inducing factor DIF-1 [1-(3,5-dichloro-2,6-dihydroxy-4-methoxyphenyl)-hexanone-1-one], (Sigma) and incubated for 30 h at 22 °C. The cells were resuspended at a density of 1 × 10^6^ cell/ mL in SB and divided into 300 µL aliquots. The aliquots were placed into test tubes containing 1 µg/mL Propidium Iodide (PI) stain, and they were incubated for 10 min at 22 °C without washing. Cytometry analysis was then performed on a FACSCalibur cytometer from Becton Dickinson using CellQuest software [16]. Experiments were performed in triplicate.

### 2.13. Determining Cell Viability Due to ATP Levels

The number of viable cells in culture was determined based on measuring the quantity of ATP, an indication of the presence of active cells [24]. The CellTiter-Glo^®^ Luminescent Cell Viability Assay kit (Promega) was used for this test. Approximately 5 × 10^5^ vegetative cells were harvested, resuspended in 100 µL of 10 mM MES (Sigma), and then transferred into opaque-walled 96-well plates [25]. Cells were lysed by adding 100 µL of the CellTiter-Glo reagent, mixed on a shaker for 2 min, and incubated at room temperature for 10 min. Microtiter^®^ Plate Illuminometer B36580 with Revelation MLX software was used to measure the ATP levels [25]. Experiments were performed in triplicate.

### 2.14. Cellular Re-Growth Assay

Vegetative cells in the late exponential growth phase were harvested and washed twice with SB. Approximately 5 × 10^5^ cells were added to 1 mL of SB containing 3 mM cAMP in a two Lab-Tek chamber and incubated for 8 h at 22 °C. The cells were washed with 1 mL of SB that was later replaced with either 1 mL of SB or 1 mL of SB containing 0.1 mM DIF-1 and incubated for 30 h at 22 °C. From each chamber, 0.5 mL of SB was removed and replaced with 1 mL of HL5 medium and incubated for 72 h at 22 °C. Cells were detached and counted using a hemocytometer and phase contrast optics. Finally, the ratio of the number of cells re-growing in the DIF-1 chamber to the number of cells in the control chamber was calculated [16]. Experiments were performed in triplicate.

### 2.15. Statistical Analysis

Statistical analysis for the endocytosis, exocytosis, phagocytosis, and membrane recycling assays was performed using by running the data through a 2-way ANOVA to test for significance at *p* < 0.01. Statistical analysis for the growth, cell size, and volume assays was performed by running the data through a student’s *t*-test with *p* ≤ 0.05. Statistical analysis for the secretion of lysosomal enzyme assays was performed using by running the data through a 2-way ANOVA to test for significance at *p* < 0.05. Statistical analysis for the chemotaxis assay was per formed by running the data through a student’s *t*-test with *p* ≤ 0.05. Statistical analysis for regrowth, cell death, and metabolic activity assays was performed by running data run through 2-way ANOVAs to test for significance at *p* < 0.05.

## 3. Results

### 3.1. Rates of Endocytosis

**Figure 1:**
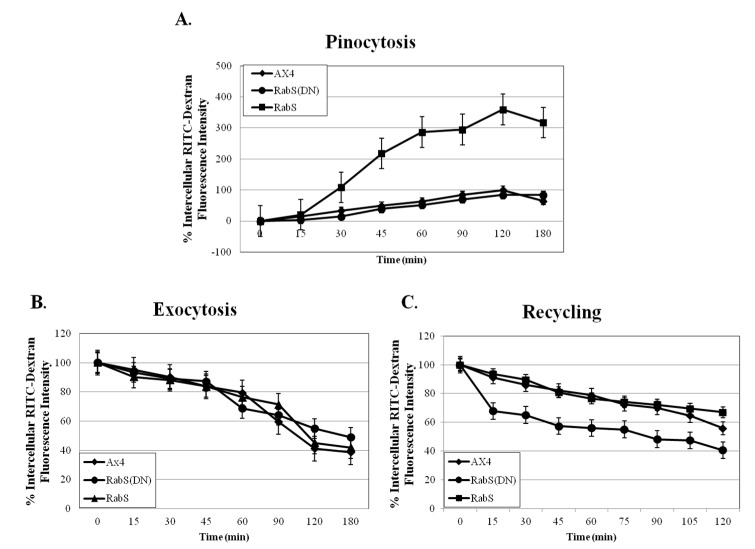
Pinocytosis, Exocytosis, and Recycling are altered in DdRabS overexpressing and DdRabS(DN) cells. (**A**) Graphical representation with standard error of the rate of pinocytosis in DdRabS overexpressing and DdRabS (DN) cell lines compared to the parent strain AX4 cells. Data are presented as relative fluorescence to AX4 which is considered 100% at 120 min. The rate of pinocytosis in the DdRabS overexpressing cell line is increased compared to AX4 cells, and the values for the pDneo2a-GFP empty vector were similar to those of the parent cell line throughout the experiments (data not shown). (**B**) Graphical representation with standard error of the rate of exocytosis. At the indicated times, the remaining intracellular RITC-dextran was measured. DdRabS overexpressing and DdRabS(DN) cells did not show a significant change in the rate of exocytosis. (**C**) Graphical representation with standard error of RITC-Dextran recycling over 120 min. DdRabS(DN) cells show an increase in the rate of recycling compared to AX4 cells. Data represent the mean of three independent experiments. Data was run through a 2-way ANOVA to test for significance at *p* < 0.01.

**Figure 2:**
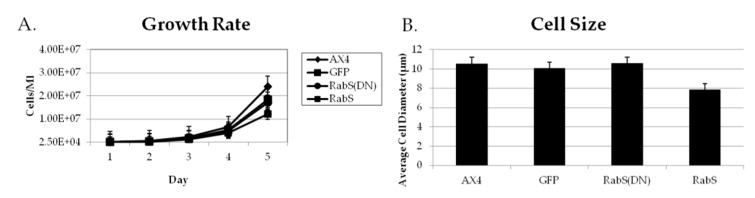
Growth rates are altered in transfected cell lines. Graphical plot of the growth rates of AX4, DdRabS, and DdRabS(DN) expressing cell lines and their sizes. (**A**) Cells overexpressing DdRabS showed an increase in the growth rate compared to AX4 cells. DdRabS(DN) showed a slight increase in growth rate compared to the WT AX4 cells. (**B**) Cells overexpressing RabS show a 26% decrease in cell diameter and a 45% decrease in cell volume. Data represent the mean of three independent experiments ±SD, as shown in Table 1. Data was run through a student’s *t*-test with *p* ≤ 0.05.

To assess the rates of endocytosis, the cells’ ability to internalize the fluid phase marker rhodamine B isothiocyanate-dextran (RITC-dextran) was measured. DdRabS overexpressing cells showed a significant increase in the internalization of RITC-dextran compared to the WT strain AX4 cells, reaching a maximum endocytic rate nearly three times that of AX4 cells after 120 min. DdRabS(DN) cells show rates similar to that of the AX4 cells (Figure 1A). These rates roughly correlated with the growth rates of these mutants in axenic culture. DdRabS overexpressing cells grew at a much faster rate than AX4 cells; they reached a titer of 1.2 × 10^7^ cells/mL after 5 days compared to 5.7 × 10^6^ cells/mL for AX4 cells while DdRabS(DN) mutants grew at a rate comparable to that of the WT. The size and volumes of the cells did not significantly differ between the AX4, pDneo2a-GFP, and RabS(DN) cell lines at 10.54, 10.06, and 10.57 µm and 87.21, 79.44, and 87.7 µm^3^ respectively; however, the RabS overexpressing cell line was significantly smaller at 7.81 µm and 47.88 µm^3^ (Figure 2). The rates of exocytosis and fluid phase recycling of RITC-dextran was also quantified; the ability of the DdRabS overexpressing and DdRabS(DN) cells to release the marker was similar to that of the AX4 cell line (Figure 1B), and the rate of fluid phase recycling of RITC-dextran was increased in DdRabS(DN) cells (Figure 1C).

### 3.2. Phagocytosis

**Figure 3:**
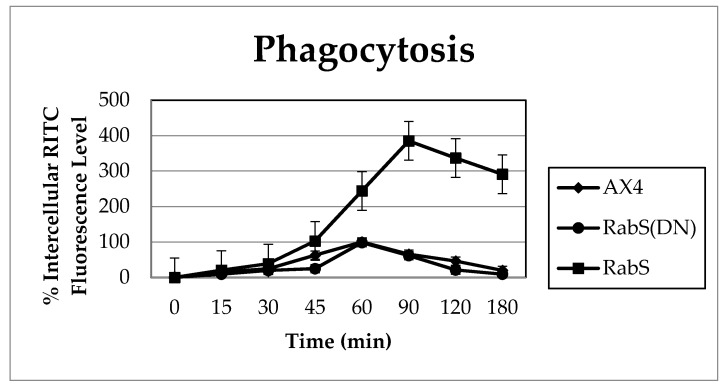
Phagocytosis is increased three-fold in cells overexpressing DdRabS vs. the WT. Graphical representation with standard error of the rate of phagocytosis over 180 min showing a significant increase in the rate of phagocytosis in DdRabS overexpressing (׀) cells over the AX4 (♦) parent strain. Data is presented as relative fluorescence to AX4, which is being considered 100% at 60 min, and the values for the pDneo2a-GFP empty vector were similar to those of the parent cell line throughout the experiments (data not shown). Data represent the mean of three independent experiments. Data was run through a 2-way ANOVA to test for significance at *p* < 0.01.

To measure the rate of phagocytosis, all cell lines were assayed for their ability to internalize latex beads. A representative experiment shown in Figure 3 indicates an increase in the internalization of latex beads in the DdRabS overexpressing cell line. After 30 min, DdRabS overexpressing cells begin to show an increase in phagocytosis, and by the 60 min time point, when the parent strain AX4 cells have a phagocytosis rate of 100%, the DdRabS overexpressing cell line has a phagocytosis rate nearly twice as high. After 90 min, DdRabS overexpressing cells reach their maximum phagocytosis rate at nearly three times that of the AX4. However, DdRabS(DN) cells phagocytize latex beads at a rate comparable to AX4 cells (Figure 3). Microscopic examination supported the internalization of the latex beads (data not shown).

### 3.3. Localization

**Figure 4:**
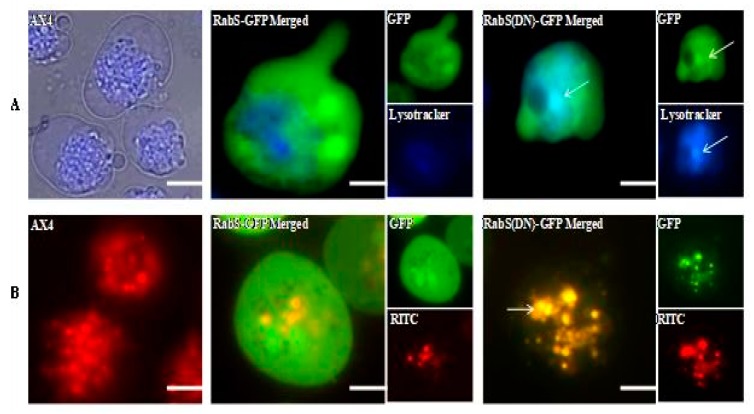
DdRabS(DN)-GFP associates with lysosomal and endosomal organelle markers. (**A**) Distribution of LysoTracker^®^ Blue dye in the AX4, DdRabS-GFP overexpressing, and DdRabS(DN)-GFP cell lines. The DdRabS-GFP overexpressing cells do not show association with the lysosomes. DdRabS(DN)-GFP cells show colocalization with the lysosomes (blue and indicated by arrows). (**B**) Distribution of RITC-dextran in the AX4, DdRabS-GFP overexpressing, and DdRabS(DN)-GFP cell lines. The vesicles of the endocytic system, from endosome to lysosome, are shown as red staining membranes due to the uptake of RITC-dextran after 60 min of treatment. The WT AX4 cells show the endosomal system in red. DdRabS-GFP overexpressing cells do not suggest colocalization with the endosomal system. DdRabS(DN)-GFP cells indicate complete colocalization with the endosomal system. These images support a role for DdRabS in vesicular trafficking. Scale bars represent 5 µm.

As DdRabS seemed to play a role in endocytosis and recycling, a microscopic approach was taken to investigate the possible localization of DdRabS to lysosomes and endosomes. Cells expressing DdRabS-GFP and DdRabS(DN)-GFP were stained with LysoTracker^®^ (Molecular Probes) which stains acidic organelles, including lysosomes, [18] or were allowed to internalize endosomal marker RITC-dextran; the cells were then photographed to determine the overlap of the GFP tagged proteins with the lysosomes or endosomes. Figure 4A (top) shows cells stained with LysoTracker^®^ blue, and Figure 4B (bottom) shows cells after the internalization of RITC-dextran. DdRabS-GFP overexpressing cells do not appear to colocalize with lysosomes or endosomes. The quantification of this colocalization supported these findings with the Pearson correlation coefficient values of −0.138 and −0.099 for DdRabS-GFP to the respective organelles. Interestingly, DdRabS(DN)-GFP cells show colocalization with both the lysosomes and the endosomes. The quantification of this colocalization supported these finding with correlation coefficient values of 0.627 and 0.768 for DdRabS(DN)-GFP, to their respective organelles. This data confirms that, while DdRabS proteins do not significantly colocalize to either lysosomes or endosomes, DdRabS(DN)-GFP proteins do. Colocalization was quantified as mentioned above.

### 3.4. L.ysosomal Secretion Assay

**Figure 5:**
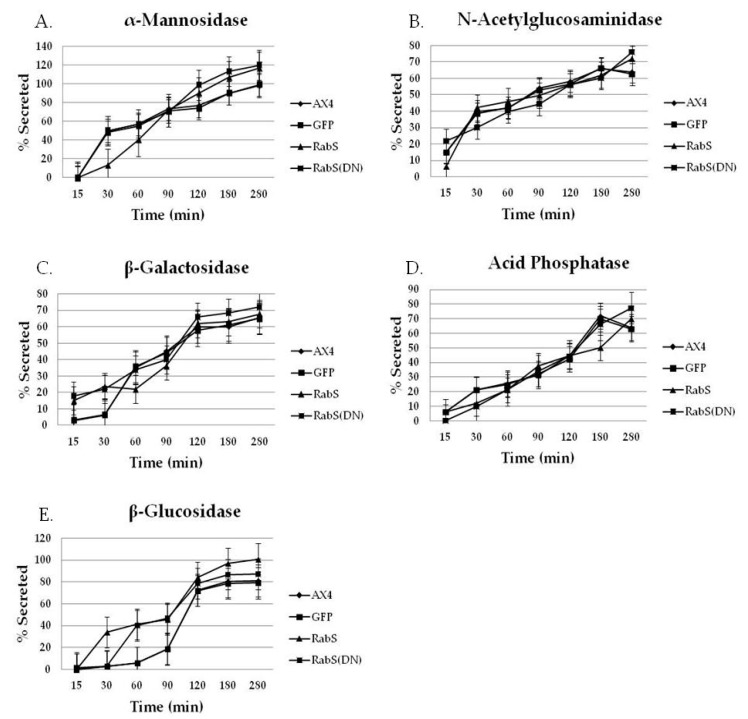
DdRabS upregulates the secretion of lysosomal glycosidase and glucosidase enzymes. Graphical representation with standard error of the secretion of the lysosomal enzyme β-glucosidase. (**A**–**E**) Logarithmically growing cells were grown under standard secretion conditions, separated into cellular and media fractions by centrifugation, lysed with Triton X-100, and each fraction was assayed for (**A**) α-mannosidase (**B**) N-acetylglucosaminidase, (**C**) β-galactosidase, (**D**) acid phosphatase activity and (**E**) β-glucosidase. Symbols are as follows: (♦) AX4, (■) pDneo2a-GFP, (▲) DdRabS, and (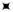) DdRabS(DN). DdRabS overexpressing and DdRabS(DN) cells show abnormal secretion of all enzymes assayed. Secretion of the lysosomal enzyme β-glucosidase is increased in DdRabS overexpressing and DdRabS(DN) cell lines. Cells overexpressing the DdRabS protein had a higher rate of secretion of β-glucosidase compared to the WT AX4 cells. DdRabS overexpressing cells reached 100% secreted enzyme after 280 min whereas the AX4 cells peaked at 80% secretion at 280 min. The DdRabS(DN) cell line has increased secretion of β-glucosidase early on, but by 120 min, secretion is only slightly more than the WT AX4 cell line. Data represent the mean of three independent experiments. Data was run through a 2-way ANOVA to test for significance at *p* < 0.05.

The role of DdRabS in the secretion of lysosomal enzymes was examined by performing standard secretion assays, as described. The extracellular (secreted) enzyme activity was expressed as a percent of total enzyme activity. All cell lines were assayed for secretion of α-mannosidase, N-acetylglucosaminidase, β-galactosidase, acid phosphatase, and β-glucosidase activity. The amounts of the secreted enzymes by the control cell lines, AX4 and pDneo2a-GFP, were approximately equal as determined by the analysis of Figure 5. Interestingly, the secretion of most of the lysosomal enzymes assayed was affected in DdRabS overexpressing and DdRabS(DN) cells. The enzyme α-mannosidase was initially secreted 40% less in DdRabS overexpressing cells than AX4 cells; however, around the 90 min time point, secretion was equal to that of the AX4 cells, and after 90 min, DdRabS overexpressing cells were secreting 10–20% more α-mannosidase than AX4 cells. On the other hand, DdRabS(DN) cells secreted α-mannosidase at a similar rate as AX4 cells up to the 90 min time point. After 90 min, DdRabS(DN) cells were also secreting 10–20% more α-mannosidase than AX4 cells (Figure 5A). The secretion of N-acetylglucosaminidase and β-galactosidase by DdRabS overexpressing and DdRabS(DN) cells was similar to AX4, but after 280 min, DdRabS overexpressing and DdRabS(DN) cells secreted approximately 10% more enzyme than AX4 cells (Figure 5B,C). The secretion of acid phosphatase was unaffected by DdRabS overexpressing and DdRabS(DN) cells (Figure 5D). Activity levels for N-acetylglucosaminidase, β-galactosidase, and β-glucosidase did not change significantly over the last few time points while levels for α-mannosidase and acid phosphatase did.

The secretion of β-glucosidase, however, was most affected. After 30 min, DdRabS overexpressing cells were secreting 30% more β-glucosidase than AX4 cells. After 60 min, this increased by 10%. After 120 min, oversecretion of β-glucosidase held steady at approximately 10–20% greater than AX4 cells. DdRabS(DN) cells also secreted more β-glucosidase, although this over-secretion does not begin until the 60 min time point; at this point, the secretion of β-glucosidase is nearly four times that of the AX4 cells (Figure 5E).

### 3.5. Development

**Figure 6:**
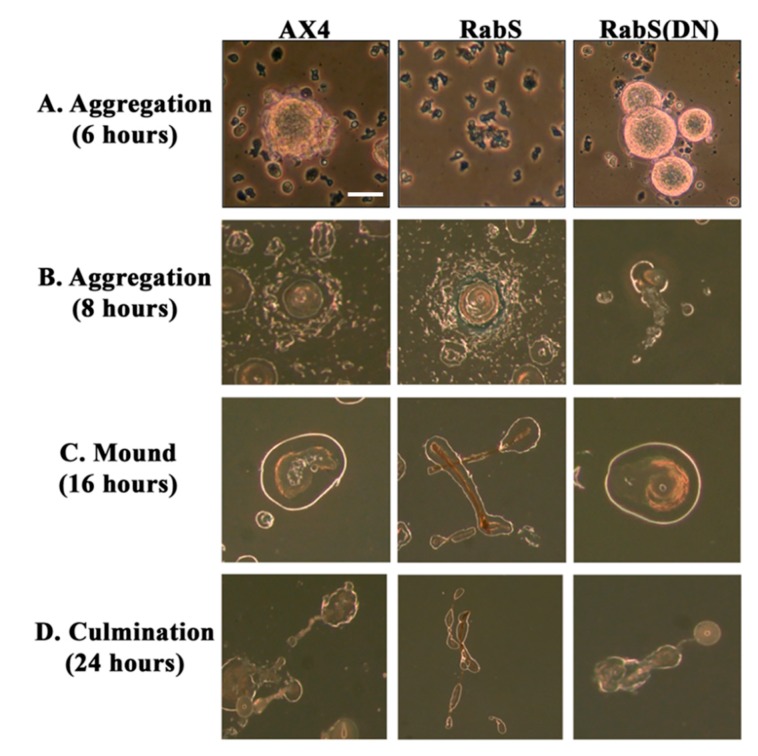
DdRabS over-expressing cells are unable to complete development. Representative photomicrographs of the phases of development in AX4, pDneo2a-GFP, RabS over-expressing, and RabS(DN) cell lines. (**A**) Aggregation after 6 h. WT AX4, and pDneo2a-GFP cells aggregated normally while the RabS overexpressing cells did not appear to aggregate. The RabS(DN) cells aggregated at a slightly faster rate than the controls. (**B**) Aggregation after 8 h. WT AX4, and pDneo2a-GFP cells aggregated normally after 8 h. RabS over-expressing cells formed very small abnormal aggregates after 8 h. RabS(DN) cells began mound formation after 8 h. (**C**) Mound formation after 16 h. AX4 and pDneo2a-GFP cells formed normal mounds after 16 h. RabS over-expressing cells appeared to have formed smaller elongated mounds compared to the controls. RabS(DN) cells formed a normal mound. (**D**) Culmination after 24 h. AX4 and pDneo2a-GFP cells formed fruiting bodies after 24 h. RabS over-expressing cells did not form fruiting bodies. RabS(DN) cells appeared to form normal fruiting bodies after 24 h. Experiments were performed in triplicate. Scale bar represents 1 mm.

Since most secretory mutants in *Dictyostelium* (roughly 80%) are unable to form cell aggregates, the prerequisite for building a mature fruiting body [26,27], the abnormal secretion of lysosomal enzymes observed in DdRabS overexpressing cells suggests they would not be able to complete development. After initiating development as described, the phases of development were observed at hours 6 and 8 (aggregation), 16 (mound formation), and 24 (culmination). Analysis of developmental processes showed that the timing and morphology of cell development in control cells expressing GFP alone was identical to that of AX4 cells. However, DdRabS overexpressing cells displayed a delayed aggregation, and after 6 h, these cells had failed to form aggregates. After 8 h, these cells had begun to form very loose, small aggregates unlike the WT cells, which were beginning to form early mound-like structures. DdRabS(DN) cells completed their developmental process; it was observed that there was an increase in the rate of aggregation after the 6 h’ time-point (Figure 6A), and after 8 h, these cells had begun to form mound structures from the aggregates (Figure 6B). After 16 h, mound formation had occurred in AX4, pDneo2a-GFP, and DdRabS(DN) cells. Interestingly, DdRabS overexpressing cells formed very deformed and elongated mound-like structures (Figure 6C). After 24 h, AX4, pDneo2a-GFP, and DdRabS(DN) cells had completed the developmental cycle with the formation of similar numbers of fruiting bodies, whereas DdRabS over-expressing cells were unable to complete development and form fruiting bodies (Figure 6D, Table 2).

### 3.6. Chemotaxis

**Figure 7:**
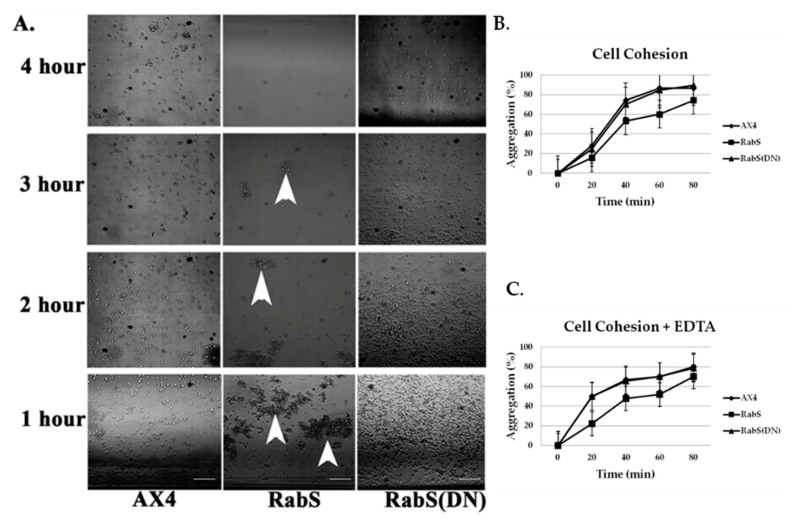
DdRabS overexpressing cells have decreased chemotactic activity in response to cAMP, and cell cohesion is decreased in DdRabS overexpressing cells. (**A**) Micrograph of chemotactically moving cells. After 4 h, the *Dictyostelium* cells moved beyond one field of view on the agarose in a 60 × 15 mm Petri dish. The amoeboid control cells, WT AX4, clearly showed trails toward the source of cAMP. Cells overexpressing RabS showed a decreased response to cAMP (arrows) while RabS(DN) cells moved towards the source of cAMP at a similar rate as WT AX4 cells. Cell cohesion was evaluated by monitoring the reassociation of cells over time without (**B**) or with (**C**) the addition of 10 mM EDTA. Cells that were not in aggregates at time zero and the percentage of cell reassociation was calculated by scoring non-aggregated cells with an hemocytometer (percentage aggregation = (total number of non-aggregating cells)/total number of cells). RabS overexpressing cells have decreased cohesion compared to the WT AX4 cells. Data represent the mean of three independent experiments. Data was run through a student’s *t*-test with *p* ≤ 0.05.

To determine if the defect in aggregation could be contributed to a defective response to cAMP, cells were assayed for their ability to respond chemotactically to a chemical gradient of cAMP. DdRabS overexpressing cells showed a delayed response to cAMP. After 4 h, DdRabS overexpressing cells had clumped together forming loose aggregates but did not migrate as far as the controls (Figure 7A).

### 3.7. Cell-Cell Adhesion

The failure to undergo development could be due to a reduction in cell-cell adhesion leading to decreased aggregation. To test this, we measured adhesitivity in the DdRabS overexpressing and DdRabS(DN) cells during development as described [21,22,23]. While reassociation occurred for all cell lines, DdRabS overexpressing cells showed a predicted decrease in adhesion (Figure 7B). To further assess adhesion, cells were assayed for EDTA-resistant cell contacts using 10 mM EDTA. During the first few hours of development, cells become gradually more cohesive, and shortly before aggregation, they display EDTA-resistant cell contacts [28]. DdRabS overexpressing cells again showed a decrease in adhesion when compared to AX4 and DdRabS(DN) cells (Figure 7C).

### 3.8. Cell Death & Viability

**Figure 8:**
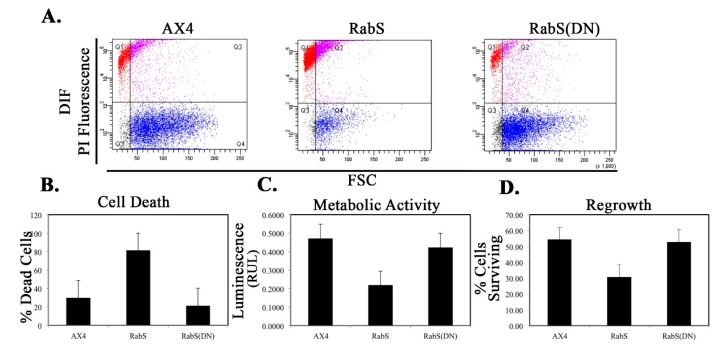
Cell death quantification using flow cytometry analysis; RabS overexpressing cells have decreased metabolic activity and regrowth ability as compared to controls. (**A**–**D**) Cell death quantification using flow cytometry analysis. (**A**) Cells were stained with 1 µg/mL Propidium Iodide, and fluorescent PI-positive cells were quantified using flow cytometry. Dot plot data with side scatter and forward scatter showed dead cells distinct from living cells. RabS over-expressing cells show an increased level of induced cellular death as compared to the WT AX4 cells. (**A**) Histogram of the dot plot data. (**B**–**D**) Graphical representation with standard error. (**B**) Quantification for DIF-1 treated cells undergoing apoptosis. RabS overexpressing cells show an increase in induced cell death. (**C**) Metabolic activity showed as a decrease in the luminescent signal from the RabS overexpressing as compared to the AX4 cell line. (**D**) Regrowth ratios of the mutant cells after 72 h of treatment with DIF-1. RabS overexpressing cell lines showed a decreased survival rate compared to the WT AX4 and pDneo2a-GFP cell lines. Data represent the mean of three independent experiments. Data was run through a 2-way ANOVA to test for significance at *p* < 0.05.

DdRabS overexpressing cells showed an increase in cell death in response to DIF-1 compared to the AX4, pDneo2a-GFP, and RabS(DN) cell lines (Figure 8A,B). Approximately 83% of the DdRabS overexpressing cells were dead while all other cell lines averaged around 25%. This could possibly be due to an increased sensitivity to DIF-1 resulting in more cells differentiating into dead stalk cells. To further quantify the cell death process, and confirm our analysis with flow cytometry, the viability of the cells was determined by measuring the amount of ATP, an indicator of cellular metabolic activity [24]. Using a luminometer, a luminescent signal relative to the amount of ATP present was measured. The luminescence signal from DdRabS overexpressing cells was greatly reduced compared to AX4, pDneo2a-GFP, and DdRabS(DN) cells, indicating that these cells are not metabolically active, confirming flow cytometry data (Figure 8C).

### 3.9. Regrowth

The survivability of cells after treatment with cAMP and DIF-1 was quantified as described by [29]. Survivability refers to the ability to multiply after the treatment. The survivability of AX4, pDneo2a-GFP, and DdRabS(DN) cells was roughly 55% of the total cells to begin with, while DdRabS overexpressing cells had approximately 30% survivability (Figure 8D).

## 4. Discussion

Upon analysis, it was found that the rates of both phagocytosis and pinocytosis were significantly increased in cells overexpressing DdRabS. Furthermore, cells expressing the DdRabS(DN) protein showed a significantly increased rate of recycling. The RabS(DN) protein may be down-regulating DdRabS signaling pathways accounting for the increased rate of recycling observed in the GDP-bound protein. Another possibility is that DdRabS may normally function to negatively regulate the process of endocytic recycling in *Dictyostelium*; this is similar to the findings [30] that DdRab11 acts to negatively regulate the rate of phagocytosis as expression of DdRab11(DN) resulted in an increase in this process. The correlation in the rates of phagocytosis, pinocytosis, and growth rate observed in DdRabS overexpressing cells has also been previously observed in the overexpression of *Dictyostelium* GTPase RacH [31], which plays a role in endocytic vesicular trafficking. The smaller size of the cells in the RabS overexpressing cell line could also contribute to the decreased rates of phagocytosis, pinocytosis, and growth.

It is possible that by overexpressing DdRabS, downstream signals triggering phagocytosis are being up-regulated, or that it may play a role in regulating the formation of the phagocytic cup or phagosome maturation. It has been proposed that formation of the phagocytic cup in many cells involves recruitment of endo-lysosomal vesicles [32,33,34]. If DdRabS is involved either directly or indirectly in the formation or maturation of the phagosomes, overexpressing it may in turn be causing more phagosomes to form resulting in the increased rate of phagocytosis observed. The fact that the DdRabS(DN) mutant cells displayed phagocytic rates similar to that of AX4 cells suggests an indirect role for DdRabS in phagocytosis. DdRabS may also be responsible for trafficking key players in the formation or maturation of phagosomes. Mutant DdRabS(DN) cells would still be able to traffic these proteins; however, they would not be able to leave the membrane after docking due to their GDP-locked form.

DdRabS has been shown to localize between the ER-Golgi membranes and the CV system [15], and it also partially colocalizes with endosomes. DdRabS(DN) cells colocalize to endosomes and lysosomes, and it has been hypothesized that DdRabS may also be involved in trafficking along the endo-lysosomal pathway as well. In this study, it was demonstrated that overexpressing DdRabS causes a significant increase in the rates of growth, phagocytosis, and pinocytosis, and that cells expressing DdRabS(DN) are defective in endocytic recycling. It has been demonstrated that DdRab2 plays a role in vesicular trafficking, and given the similar locations of DdRab2 and DdRabS, this prompted us to investigate a possible role for DdRabS along the endo-lysosomal pathway [35].

Both DdRabS overexpressing and DdRabS(DN) cells exhibited an up-regulation in the secretion of lysosomal enzymes, with the greatest change being observed in the oversecretion of β-glucosidase. DdRabS is deficient in development, cell-cell adhesion, chemotaxis, regrowth, and viability, which could be related to the increased release of lysosomal enzymes. However, it shows an increase in cell death in response to DIF-1. This data suggests that the DdRabS protein functions in the transport of proteins from the ER-Golgi-CV as well as along the endo-lysosomal pathway, and that after docking with the membrane, the GDP-locked DdRabS(DN) is not able to leave the membrane accounting for the differential localization observed. Under normal conditions, DdRabS may only be dropping its cargo to the endosomes and lysosomes and then continuing on. This would explain why DdRabS-GFP does not show localization with these organelles in the overexpressing cell line. It is not able to accumulate on the membranes as in the DN form. This microscopic evidence coupled with the graphical evidence suggests a role for DdRabS along the endo-lysosomal pathway [35].

Normally, Rab proteins are activated by the exchange of GDP for GTP, which is triggered by guanine nucleotide exchange factors (GEFs). Once an individual transport step is completed, GTPase-activating proteins (GAPs) accelerate Rab GTP hydrolysis, allowing recognition by a GDP dissociation inhibitor (GDI), which sequesters the Rab in the cytosol until it is recruited to a membrane and begins the transport cycle again [6,36]. We propose that DdRabS(DN) is unable to leave the endosomal and lysosomal membranes because it is locked in its GDP-bound state preventing it from converting to the GTP-bound form after delivering its cargo; thus, it accumulates in these organelles accounting for its differential localization.

Statistically significant differences were found in the secretion of lysosomal enzymes, both between the cell lines and the time points samples were taken at. After following standard secretion conditions [19], more than 50% of the total cellular activity of several lysosomal glycosidases was secreted within a few hours; these conditions are particularly useful because the lysosomal enzymes are secreted in the absence of any appreciable enzyme synthesis or degradation. Thus, by monitoring the release of lysosomal enzymes, the functioning of the lysosomal vesicles involved in secretion can be observed [19]. As compared to all tested cell lines, we observed that DdRabS mutants slightly oversecrete α-mannosidase, N-acetylglucosaminidase, and β-galactosidase and significantly oversecrete β-glucosidase. The secretion of acid phosphatase appeared unaffected.

It has been proposed that there are three functional classes of lysosomal vesicles in *Dictyostelium;* two of these classes are secretory, and the other is not. While acid phosphatase is believed to belong to one of the secretory classes, α-mannosidase, N-acetylglucosaminidase, β-galactosidase, and β-glucosidase are believed to belong to the other [19,37,38]. Another possibility presented by the Cardelli group [37] is that acid phosphatase secretion is differentially regulated compared to the glycosidases. Our results indicate that DdRabS is involved with the class of lysosomal vesicles containing glycosidases as the secretion of acid phosphatase appeared unaltered when compared to the control cell lines. The secretion of β-glucosidase was highest in DdRabS overexpressing cells and while this phenotype was rescued slightly by DdRabS(DN) cells, the cells still oversecreted the enzyme. These results are consistent with a role for DdRabS in the secretion of lysosomal enzymes. The oversecretion observed may be related to improper acidification of endosomes. It was indicated in previous reports on *Acanthamoeba* that hydrolase transport and secretion is pH dependent [31,39]. Starvation initiates the developmental cycle in *Dictyostelium*, which is, in part, characterized by induced secretion of lysosomal hydrolases relative to secretion during growth [10].

Regulated secretion is also characteristic of *Dictyostelium* development; when cells reach high density and during early development, they secrete lysosomal hydrolases [3,10,13], cell-density sensing factor (CMF) [3,40,41,42] and cell-counting factor (CF) [3,43,44,45,46,47], which can regulate aggregate size during development. Countin, a subunit of CF, negatively regulates adhesion by regulating the expression of CAMs [44]. In mutants that over-secrete countin, there is decreased adhesion, and in mutants where countin is disrupted (countin 2), there is increased adhesion [44]. It is possible that overexpressing DdRabS also causes an oversecretion of the lysosomal hydrolase countin accounting for the decrease in adhesion; it is also feasible that this oversecretion could be related to the elevated rate of death the RabS cell line.

In the developmental assay, DdRabS overexpressing cells failed to complete development; the cells were unable to aggregate normally and did not develop fruiting body structures. DdRabS overexpressing cells were also less cohesive than AX4 cells at both the EDTA-sensitive and EDTA-insensitive contact sites. DdRabS overexpressing cells also displayed a decreased sensitivity and migratory response to the chemoattractant cAMP, and they showed an increased sensitivity to DIF-1 causing an increase in induced cell death. In DdRabS(DN) cells, all of these phenotypes were rescued. We propose that the overexpression of DdRabS interferes with the function of other small GTPase proteins crucial for these processes to proceed normally.

Overexpression of DdRabS caused very abnormal chemotaxis. Chemotaxis was not completely abolished as cells did migrate slightly and cluster together forming a loose type of aggregate. However, their migration towards the cAMP was minimal. This could be due to the cells being unable to detect cAMP or to the cells being unable to migrate towards the source of cAMP. Binding of cAMP causes dissociation of the heterotrimeric G protein. This in turn causes activation of small GTP-binding proteins of the Ras family [48,49]. It is possible that by overexpressing DdRabS, GTP-binding proteins of the Ras family are unable to be activated. As stated previously, some GDP/GTP exchange proteins (GEP), GAPs, and GDIs, which are crucial for GTPase activation, show wide substrate specificities [49,50,51]. It is possible that these GTP-activating proteins are binding with DdRabS, leaving them unavailable to activate the Ras family GTPases that are crucial for the signaling cascade of chemotaxis. During our investigation, we observed a possible role for DdRabS in phagocytosis in the formation of the phagocytic cup. F-actin is essential for the formation of the phagocytic cup and is also an integral member of the actin cytoskeleton [34]. It is possible that DdRabS may play a role in the regulation of F-actin, which would explain the phenotypes observed in both phagocytosis and motility. This is supported by the suggestion of [34] that there is a connection between the actin cytoskeleton and membrane trafficking.

Cell-cell contact, as well as cAMP, plays a key role in cellular differentiation and gene expression in *Dictyostelium*. Several studies have suggested that cell-cell contact has a major influence on the expression of many developmentally regulated genes [21]. In cells overexpressing DdRabS, cell–cell adhesion is reduced in both EDTA-sensitive, and EDTA-insensitive contacts. Previous reports have implicated Ras family proteins as being crucial for downstream signaling to adhesion molecules DdCAD-1 and gp80 [21,23]; some GEP, GAP, and GDI that are critical for Ras GTPase activation show a wide substrate specificity [49,50,51] and will bind to several Rab isoforms [52]. It is possible that through overexpressing DdRabS, binding proteins required for Ras activation are instead bound to DdRabS causing Ras to remain inactive resulting in neither DdCAD-1 nor gp80 being signaled. These results indicate that overexpressing DdRabS may have an effect on both DdCAD-1 (EDTA-sensitive contacts) and gp80 (EDTA-insensitive contacts), possibly by affecting GTPase proteins in charge of signaling these adhesion molecules. This would explain the difficulty DdRabS overexpressing cells have with the aggregation process. The fact that the DN mutant does not show decreased cohesion supports this as it is locked in its GDP-bound form leaving the GEP’s available for Ras activation.

We tested the effects of DdRabS overexpression on induced cell death by incubating cells first with cAMP then DIF-1 as described above. DdRabS overexpressing cells showed a significant increase in autophagic induced cell death as compared to the controls. These cells also had decreased ATP levels and were unable to re-grow after DIF-1 treatment; this confirmed that these cells have died. Our data showed that although DdRabS overexpressing cells showed great difficulty aggregating, they did eventually form small loose aggregates that later gave rise to abnormal, elongated, small mound structures. The two types of prestalk cells, prestalk A (pstA) and prestalk B (pstB), are first detectable in the *Dictyostelium* mound. pstB cells are found at the base and are sensitive to cAMP, and pstA cells are in the apex and form the tip of the stalk. In the presence of cAMP, pstB cells are more slowly induced by DIF-1 [53]. It is possible that DdRabS overexpressing cells, which may have a decreased sensitivity to cAMP, possess a greater number of pstB cells being induced by DIF-1. The base of the stalk would then form. However, it would not actually form the stalk or spore head. This theory supports our data finding that DdRabS overexpressing cells do not form fruiting body structures and are very sensitive to induced autophagic cell death by DIF-1.

## 5. Conclusions

The results of this work show an increased rate of phagocytosis and pinocytosis as well as differential localization to endosomes and lysosomes in DdRabS(DN) cells. DdRabS overexpressing and DdRabS(DN) cells also demonstrate oversecretion of lysosomal enzymes—specifically, the oversecretion of glucosidases. While it is clear that overexpressing DdRabS has an adverse effect on *Dictyostelium* development, the exact mechanisms being disrupted are unclear. One possibility to further explore includes loose substrate specificity for GTPase switch molecules that DdRabS may be using; this prevents development essential GTPases from becoming activated resulting in a cascade of negative effects throughout development. Due to its effects on osmoregulation, it is hypothesized that DdRabS may be trafficking vacuolar proton pumps from the ER to the Golgi where they are modified and are then mainly transported to the CV along the endo-lysosomal or secretory pathway [15]. We further hypothesize that overexpression of DdRabS may interfere with GTP activation of other GTPase related proteins essential for normal development in *Dictyostelium* resulting in a cascade of defects throughout this process. Due to the lack of a clearly distinctive effect for the DdRabS(DN) protein, the possibility of a redundancy in function for this protein must be considered: there is the possibility that other Rab proteins possess the same function as DdRabS. It is also possible that the levels of endogenous DdRabS are high enough to mask the effect of the dominant negative mutant. Another possibility is that this mutant could be unstable and is being degraded before its function is evident. Future studies will be required to elucidate the mechanism behind this.

## Figures and Tables

**Table 1:** Comparisons in mean cell diameter ± SD and area.

	Cell Diameter (µm)	Cell Area (µm^2^)	S.D.
AX4	10.54	87.21	± 1.75
GFP	10.06	79.44	± 1.50
RabS	7.81	47.88	± 2.09
RabS (DN)	10.57	87.7	± 1.70

**Table 2:** Achievement of Developmental Milestones.

Cell Line	Aggregation (6 h)	Aggregation (8 h)	Mound (16 h)	Culmination (24 h)	Fruiting Bodies
AX4	Normal	Normal	normal	normal	115 ± 2.08
GFP	Normal	Normal	normal	normal	112 ± 2.65
RabS	No	Small	small	no	0 ± 0.00
RabS(DN)	accelerated	accelerated	normal	normal	111 ± 3.78
